# Controlled compression, amplification and frequency up-conversion of optical pulses by media with time-dependent refractive index

**DOI:** 10.1515/nanoph-2022-0818

**Published:** 2023-03-09

**Authors:** Alexander Gabriel Löhr, Misha Yu Ivanov, Margarita A. Khokhlova

**Affiliations:** Max Born Institute for Nonlinear Optics and Short Pulse Spectroscopy, Berlin, Germany; Department of Physics, Humboldt University, Berlin, Germany; Blackett Laboratory, Imperial College London, London, UK; Department of Physics, King's College London, London, UK

**Keywords:** amplification and up-conversion, laser pulse control, molecular modulators, pulse compression, Raman scattering, time-varying refractive index

## Abstract

Control over the time dependence of the refractive index of a material allows one to modify and manipulate the properties of light propagating through it. While metamaterials provide a promising avenue in this context, another route has been extensively explored by the ultrafast community — the so-called molecular modulators. Indeed, impulsively-aligned diatomic molecules provide a unique medium, where periodic rotational revivals induced by a pump pulse persist for tens of picoseconds, offering an excellent opportunity for the controlled modification of the refractive index and, therefore, of femtosecond laser pulses propagating through these media. Here we present an analytical theory which describes this process and stumble across a novel mechanism revealing exponential transformations of the probe pulse — its compression, amplification and frequency up-conversion. In particular, our analytical results predict the generation of amplified ultrashort (about 20 fs) ultraviolet pulses centered around 550 nm, starting with near infrared input pulses centered on 1 μm of about 30 fs duration, under very realistic experimental conditions.

## 1 Introduction

Electro-optic and acousto-optic modulators are some of the standard examples of using temporal control over the refractive index of a material to modify and manipulate the properties of light propagating in such materials, see e.g. [1] for a pertinent example and [2, 3] for a more general description of electromagnetic waves interacting with time-varying media. Similarly, spatial control over the refractive index allows one to tailor the medium dispersion, including generation of energy bandgaps. In this context, metamaterials have emerged as a natural medium for designing the spatially-dependent refractive index. If combined with sharp temporal modulations of the refractive index, metamaterials provide a wide array of novel physical phenomena such as spatio-temporal photonic time crystals, where time- and space-periodic reflections at spatio-temporal interfaces result in energy and momentum bandgaps [4, 5]. One of the most spectacular manifestations of photonic time crystals is exponential amplification of desired spatio-temporal modes of these crystals [6].

A practical challenge in realizing temporal photonic crystals displaying a significant momentum bandgap is the requirement of substantial and rapid temporal modulation of the refractive index needed to generate the reflected wave at a well-defined instant in time. Indeed, generating such temporal reflection requires changes in the refractive index both within a cycle of a propagating wave and on the value scale of the refractive index itself. This challenge is particularly acute in the optical regime.

In this article we focus on a separate class of spatio-temporal modulations which, while not leading to momentum bandgaps, can nevertheless result in efficient exponential transformation of the intensity, carrier frequency and duration of optical pulses. These modulations can be readily realized in abundant media such as molecular gases at ambient conditions pumped by widely available laser pulses.

We suggest a pump-probe scenario in which a strong pump induces a spatio-temporal refractive index modulation by inducing coherent rotations in the molecular medium. Subsequently, a weak probe propagates in the modulated refractive index landscape created by the pump. We find that the modulation of the refractive index enforces the amplitude, frequency and duration of the probe to change during its propagation through the medium in an exponential fashion. For the probe placed on a negative (positive) slope of the refractive index, the front and the rear of the probe pulse move with different speeds, leading to pulse compression (stretching) with blue (red) shift and its amplification (suppression). During this process the number of photons in the probe is conserved [7], as has been also understood in the context of electro-optical modulation [1].

Our theoretical analysis is based on the solution of the wave equation for the probe pulse which includes the description of dispersion in media where the refractive index depends on both frequency and time simultaneously. Our results not only support the recent findings of Pendry and co-workers [8, 9] of a new gain mechanism in time-dependent media, but also extend this analysis by treating medium dispersion, neglected in [8], and provide concrete results of the probe pulse modification due to the propagation in the medium under standard experimental conditions.

The gain mechanism we introduce comes into play already for gentle modifications of the refractive index *n*, with the modulation amplitude Δ*n* ≪ 1, and the modulation window much longer than the optical cycle of the amplified probe wave. Such modulation can be achieved in a hollow-core fiber filled with molecular gas, e.g., nitrogen.

## 2 Model

**Figure 1:**
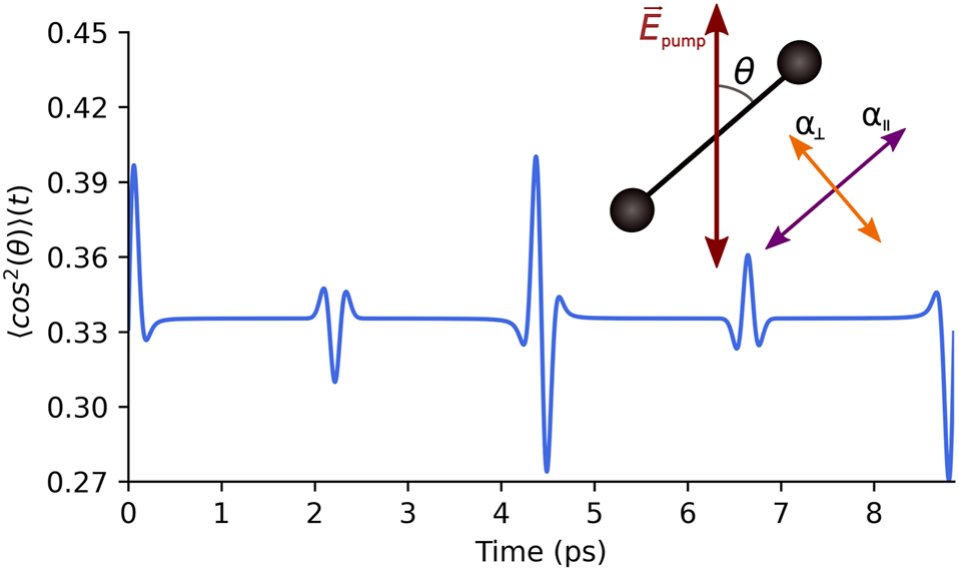
Ensemble-averaged time-dependent alignment ⟨cos^2^(*θ*)⟩(*t*) of nitrogen molecules after the pump pulse. Insert shows a schematic of a linear molecule in a linearly-polarized pump field **E**_pump_(*t*). The difference in the parallel *α*_‖_ and perpendicular *α*_⊥_ polarizabilities results in an aligning torque towards the laser polarization axis.

We consider an intense linearly-polarized infrared (IR) pump pulse interacting with a nitrogen molecule. It polarizes the electronic cloud predominantly along the molecular axis, inducing a dipole moment which follows the oscillations of the laser field. This results in a non-zero cycle-average torque exerted onto a molecule, which tends to align its axis along the laser polarization [10], see Figure 1. For a pump pulse intensity 3.3 × 10^13^ W/cm^2^ the effective aligning potential is 31 meV deep, exceeding the thermal energy at room temperature. At the same time, this intensity is insufficient for ionization of the molecule. For a pump pulse much shorter than the rotational period of the molecule, the molecular ensemble continues to rotate for tens of picoseconds after the end of the pump pulse. Importantly, the alignment of molecules triggered by the pump does not completely disappear after the end of the pump pulse. On the contrary, the coherently rotating molecular ensemble experiences periodic revivals of the overall alignment [11–13] as shown in Figure 1.

As a result of this time-dependent molecular alignment, a probe pulse, sent in the wake of the pump, experiences periodic modulations of the refractive index [14–17]:

(1) $$n\left(t\right)=n_{0}+\Delta n\left\langle \cos^{2}\left(\theta \right)\right\rangle \left(t\right),$$

where the modulation depth Δ*n* of the gaseous medium is of the order of the difference *n*_0_ − 1, where *n*_0_ ∼ 1 is the refractive index of the unperturbed molecular gas.

A similar effect is observed in a single pulse if the pulse is long enough for its rear to experience the molecular rotations induced by the front [18, 19] which leads to significant spectral broadening of the pulse.

Note that molecular rotations are not the only way to modulate the refractive index, with coherently driven molecular vibrations providing another spectacular opportunity for spectral broadening and pulse modification [20–22]. The formalism we introduce can be easily adapted to cover pulse propagation through coherently vibrating molecules by incorporation the time dependence of the refractive index introduced by the collective oscillating motion of the molecules.

A closer look at Eq. (1) reveals that the dispersion of the medium is not included. A temptation to simply insert the explicit frequency dependence by adding it to *n*_0_ and Δ*n* appears to run afoul of the fact that time and frequency are Fourier conjugate variables. Fortunately, we find below that this problem is resolved by properly treating dispersion in the time domain and separating the timescales of the attosecond electronic response and the picosecond alignment process.

## 3 Theory

**Figure 2:**
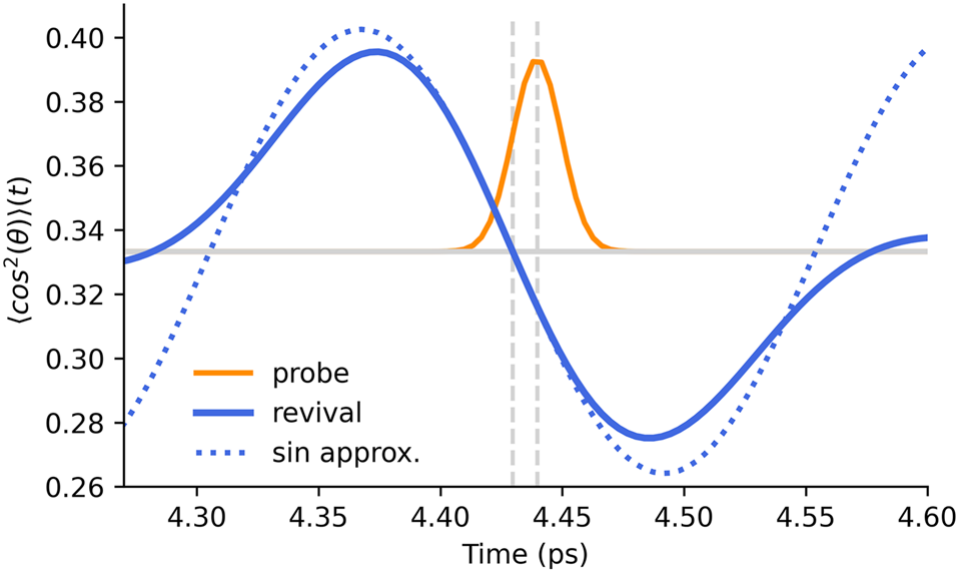
The probe pulse (orange solid line) co-propagating with the odd rotational revival (blue solid line). The centers of the probe pulse and the revival are shown with vertical gray lines indicating the relative delay *t*_0_. The probe only experiences the modulation in its immediate space-time vicinity making little to no distinction between the actual revival and the sinusoidal approximation of the revival (blue dotted line).

Based on our model, we consider a short probe pulse following in the wake of the pump, with the probe duration shorter or on the scale of about 100 fs and a pump-probe delay around 4.2 ps, see Figure 2, where the refractive index experiences a clear modulation Δ*n*(*t*). Taking into account that Δ*n* ≪ 1 and that the probe is short and weak, we can safely assume that it experiences weak reflection and its induced alignment is negligible compared to the strong pump. This allows one to treat the propagation of the probe accounting only for the linear response of the rotating molecular medium to it.

Given the separation of the time scales of the electronic and rotational response, the linear polarization **P**(*x*, *t*) induced by the probe **E**(*x*, *t*) is

(2) $$\begin{array}{ll} P\left(x,t\right) & =〈\cos^{2}\theta 〉\left(x,t\right)\overset{\infty }{\underset{-\infty }{\int }}\chi_{\|}\left(\tau \right)E\left(x,t-\tau \right)d\tau \\ & \;+〈\sin^{2}\theta 〉\left(x,t\right)\overset{\infty }{\underset{-\infty }{\int }}\chi_{⊥}\left(\tau \right)E\left(x,t-\tau \right)d\tau , \end{array}$$

where *χ*_⊥_ and *χ*_‖_ are the electrical susceptibilities of the molecule perpendicular and parallel to the molecular axis which define the fast electronic response to the probe field, while ⟨cos^2^ *θ*⟩ and ⟨sin^2^ *θ*⟩ measure the ensemble-averaged alignment of the rotating molecules along the polarization of the probe field. Note that including the slow time-varying alignment in Eq. (2) multiplicatively is an adiabatic approximation with respect to the ultrafast electronic response.

For a randomly-oriented molecular ensemble the molecular alignment is constant at ⟨cos^2^ *θ*⟩ = 1/3 and the above Eq. (2) yields the linear response of the unperturbed medium

(3) $$P_{0}\left(x,t\right)=\overset{\infty }{\underset{-\infty }{\int }}\chi_{0}\left(\tau \right)E\left(x,t-\tau \right)d\tau ,$$

with the *χ*_0_ being the electric susceptibility of the unperturbed medium

(4) $$\chi_{0}\left(\tau \right)=\left[\frac{1}{3}\chi_{\|}\left(\tau \right)+\frac{2}{3}\chi_{⊥}\left(\tau \right)\right].$$

The linear response experienced by the probe can be written as **P** = **P**_0_ + Δ**P**, where **P**_0_ is the polarization of the unperturbed medium (3), while

(5) $$\begin{array}{cc} & \Delta P\left(x,t\right)=f\left(x,t\right)\overset{\infty }{\underset{-\infty }{\int }}\Delta \chi \left(\tau \right)\;E\left(x,t-\tau \right)d\tau ,\\ & f\left(x,t\right)=\left\langle \cos^{2}\theta \right\rangle \left(x,t\right)-1/3,\\ & \Delta \chi \left(\tau \right)=\chi_{\|}\left(\tau \right)-\chi_{⊥}\left(\tau \right). \end{array}$$

Here Δ*χ*(*τ*) describes alignment-dependent anisotropy in the attosecond electronic response of a molecule to the probe field **E**(*x*, *t*) scaled with the density of the molecular gas, and *f*(*x*, *t*) defines the spatio-temporal modulation function of the response due to slow molecular rotations reflecting the deviations in the value of the molecular alignment from the unperturbed random distribution of molecules. In the frequency domain the polarization (2) becomes

(6) $$\begin{array}{ll} \tilde{P}\left(x,\omega \right) & ={\tilde{P}}_{0}\left(x,\omega \right)+\Delta \tilde{P}\left(x,\omega \right)\\ & ={\tilde{\chi }}_{0}\left(\omega \right)\tilde{E}\left(x,\omega \right)+\overset{\infty }{\underset{-\infty }{\int }}\tilde{f}\left(x,\omega -\Omega \right)\Delta \tilde{\chi }\left(\Omega \right)\\ & \;\times \tilde{E}\left(x,\Omega \right)d\Omega . \end{array}$$

Here we find that the multiplicative temporal modulation function *f* affects the polarization through a convolution in the frequency domain.

Assuming that the pump pulse moves through the medium at a constant speed, corresponding to its group velocity *v*_pump_, without absorption and without deformation due to dispersion or self-action, *f*(*x*, *t*) depends only on the delay between the pump and the probe pulse:

(7) $$f\left(x,t\right)=f\left(t-x/v_{\text{pump}}\right),$$

so that its temporal Fourier transform is

(8) $$\tilde{f}\left(x,\omega \right)=\tilde{f}\left(\omega \right){\text{e}}^{\text{i}\frac{x}{v_{\text{pump}}}\omega }.$$

We now substitute these expressions into the wave equation for the probe pulse in the frequency domain:

(9) $$\frac{\partial^{2}\tilde{E}\left(x,\omega \right)}{\partial x^{2}}+\frac{\omega^{2}}{c^{2}}\tilde{E}\left(x,\omega \right)=-\frac{4\pi \omega^{2}}{c^{2}}\tilde{P}\left(x,\omega \right).$$

For the sake of simplicity, we consider propagation of the probe pulse in one dimension (1D). Writing the spectral electric field of the probe as

(10) $$\tilde{E}\left(x,\omega \right)=\tilde{A}\left(x,\omega \right){\text{e}}^{\text{i}\frac{n\left(\omega \right)}{c}\omega x},$$

and inserting it together with Eq. (8) into the 1D Eq. (9) yields:

(11) $$\begin{array}{ll} & \frac{\partial^{2}\tilde{A}\left(x,\omega \right)}{\partial x^{2}}+2\text{i}\frac{n\left(\omega \right)}{c}\omega \frac{\partial \tilde{A}\left(x,\omega \right)}{\partial x}\\ & \;=-\frac{4\pi \omega^{2}}{c^{2}}{\text{e}}^{\text{i}\left[\frac{1}{v_{\text{pump}}}-\frac{n\left(\omega \right)}{c}\right]\omega x}\int_{-\infty }^{\infty }{\text{e}}^{\text{i}\left[\frac{n\left(\Omega \right)}{c}-\frac{1}{v_{\text{pump}}}\right]\Omega x}\\ & \;\;\times \tilde{f}\left(\omega -\Omega \right)\left[\Delta \tilde{\chi }\left(\Omega \right)\tilde{A}\left(x,\Omega \right)\right]d\Omega . \end{array}$$

Here $\tilde{A}\left(x,\omega \right)$ is the spectral envelope of the probe and *n*(*ω*) is the time-independent refractive index of the unperturbed medium associated with polarization *P*_0_ through $n^{2}\left(\omega \right)=1+4\pi {\tilde{\chi }}_{0}\left(\omega \right)$ .

Equation (11) describes the 1D evolution of the spectral envelope $\tilde{A}\left(x,\omega \right)$ as it propagates along the wake of the pump.

The convolution integral in Eq. (11) already contains two important pieces of information. First, it describes the Stokes and anti-Stokes frequency shifts imparted on the probe by the pump-induced modulations of the medium, as new frequencies are continuously generated via the convolution of the probe with the modulation function $\tilde{f}\left(\omega \right)$ (8), which is spectrally narrow compared to the probe. Second, it shows that the standard phase-matching condition is modified by the group velocity of the spatio-temporal modulation. We shall see below that it is the group velocity of the probe, and not its phase velocity, that needs to match the group velocity of the pump for optimal modification of the probe.

Equation (11) can be further simplified by assuming weak reflection of the probe from the pump-induced modulation, which corresponds to neglecting the second-order spatial derivative of the spectral amplitude, leading to

(12) $$\begin{array}{ll} \frac{\partial \tilde{A}\left(x,\omega \right)}{\partial x} & =\text{i}\frac{2\pi \omega }{n\left(\omega \right)c}{\text{e}}^{-\text{i}\left[\frac{n\left(\omega \right)}{c}-\frac{1}{v_{\text{pump}}}\right]\omega x}\int_{-\infty }^{\infty }{\text{e}}^{\text{i}\left[\frac{n\left(\Omega \right)}{c}-\frac{1}{v_{\text{pump}}}\right]\Omega x}\\ & \;\times \tilde{f}\left(\omega -\Omega \right)\left[\Delta \tilde{\chi }\left(\Omega \right)\tilde{A}\left(x,\Omega \right)\right]d\Omega . \end{array}$$

In principle, one may already use Eq. (12) for computational purposes. We have benchmarked this equation against experimental measurements in Ref. [17] with excellent agreement, see Supplementary material. However, the convolution integral over all frequencies in Eq. (12) needs to be executed at every single propagation step, slowing down the simulations unnecessarily. Giving some thought to the underlying physical picture of the short probe co-propagating with the pump-induced modulation, one can quickly realize that an accurate depiction of the modulation function is only necessary in the immediate proximity of the probe pulse, where its field is non-zero.

Taking a closer look at the temporal shape of an individual rotational revival in Figure 2, one can readily see that a sine wave is an adequate approximation if the short probe pulse is situated in the vicinity of the revival center.

Approximating the modulation function as *f* = *a*_*f*_ sin(*ω*_*f*_ *t*), we write its frequency-domain counterpart as

(13) $$\tilde{f}\left(\omega \right)=\frac{a_{f}}{2}\left[\delta \left(\omega +\omega_{f}\right)-\delta \left(\omega -\omega_{f}\right)\right],$$

where *a*_*f*_ and *ω*_*f*_ are the amplitude and frequency of the sinusoidal approximation, respectively. Inserting (13) into the propagation Eq. (12), we obtain

(14) $$\begin{array}{ll} \frac{\partial \tilde{A}\left(x,\omega \right)}{\partial x} & =\text{i}\frac{a_{f}\pi \omega }{n\left(\omega \right)c}{\text{e}}^{-\text{i}\left(\frac{n\left(\omega \right)}{c}-\frac{1}{v_{\text{pump}}}\right)\omega x}\left[\Delta \tilde{\chi }\left(\omega +\omega_{f}\right)\right.\\ & \;\times \tilde{A}\left(x,\omega +\omega_{f}\right){\text{e}}^{\text{i}\frac{n\left(\omega +\omega_{f}\right)}{c}\left(\omega +\omega_{f}\right)x}{\text{e}}^{-\text{i}\frac{\omega +\omega_{f}}{v_{\text{pump}}}x}\\ & \;-\Delta \tilde{\chi }\left(\omega -\omega_{f}\right)\tilde{A}\left(x,\omega -\omega_{f}\right){\text{e}}^{\text{i}\frac{n\left(\omega -\omega_{f}\right)}{c}\left(\omega -\omega_{f}\right)x}\\ & \;\times \left.{\text{e}}^{-\text{i}\frac{\omega -\omega_{f}}{v_{\text{pump}}}x}\right], \end{array}$$

thereby reducing the convolution integral to a sum of two terms. Eq. (14) is clearly computationally preferable over Eq. (12) and is used for our numerical simulations.

As for the analytical calculations, we make further approximations. As long as the short probe pulse is confined to the central part of the slope of the rotational revival, then the width of the spectral envelope $\tilde{A}\left(x,\omega \right)$ is considerably larger than the frequency *ω*_*f*_ associated with the rotational modulation. This allows us to replace a finite difference in (14) with a derivative

(15) $$\begin{array}{ll} \frac{\partial \tilde{A}\left(x,\omega \right)}{\partial x} & =\text{i}\frac{2\pi a_{f}\omega_{f}\omega }{n\left(\omega \right)c}{\text{e}}^{-\text{i}\left(\frac{n\left(\omega \right)}{c}-\frac{1}{v_{\text{pump}}}\right)\omega x}\\ & \;\times \frac{\partial }{\partial \omega }\left[\Delta \tilde{\chi }\left(\omega \right)\tilde{A}\left(x,\omega \right){\text{e}}^{\text{i}\left(\frac{n\left(\omega \right)}{c}-\frac{1}{v_{\text{pump}}}\right)\omega x}\right], \end{array}$$

where the product *a*_*f*_ *ω*_*f*_ is the slope of the sinusoidal temporal modulation. Thus, our approximation corresponds to a linear approximation of the sinusoidal modulation, a justifiable assumption in the vicinity of the strongly-sloped revival center. Performing the differentiation in Eq. (15), we obtain

(16) $$\begin{array}{ll} \frac{\partial \tilde{A}\left(x,\omega \right)}{\partial x} & =i\frac{2\pi a_{f}\omega_{f}\omega }{n\left(\omega \right)c}\left[\Delta \tilde{\chi }\left(\omega \right)\frac{\partial \tilde{A}\left(x,\omega \right)}{\partial \omega }-\tilde{A}\left(x,\omega \right)\right.\\ & \;\times \left\{\text{i}\Delta \tilde{\chi }\left(\omega \right)\left(\frac{n\left(\omega \right)+\frac{\partial n\left(\omega \right)}{\partial \omega }\omega }{c}-\frac{1}{v_{\text{pump}}}\right)x\right.\;\left.\left.+\frac{\partial \Delta \tilde{\chi }\left(\omega \right)}{\partial \omega }\right\}\right]. \end{array}$$

We further approximate the refractive index in Eq. (16) as *n*(*ω*) ≈ *n*(*ω*_0_) + *n*′(*ω* − *ω*_0_), with $n^{\prime }={\left.\frac{\partial n\left(\omega \right)}{\partial \omega }\right|}_{\omega_{0}}$ , while neglecting the dispersion of the susceptibility $\Delta \tilde{\chi }\left(\omega \right)\approx \Delta \tilde{\chi }\left(\omega_{0}\right)$ , where *ω*_0_ is the initial carrier frequency of the probe pulse. This approximation means that we neglect the dispersion associated with the modulated fraction of the refractive index. The frequency dispersion experienced by the probe pulse is therefore equivalent to that of an unperturbed medium, making this approximation adequate as long as the modulation depth of the response is small compared to the total response. Lastly, as we deal with gaseous media which are optically-driven far off any resonances, where the refractive index *n*(*ω*) is close to 1 [23], we can substitute *n*(*ω*) = 1 in the prefactor on the right-hand side of Eq. (16).

Applying all the aforementioned assumptions to Eq. (16) yields

(17) $$\begin{array}{ll} \frac{\partial \tilde{A}\left(x,\omega \right)}{\partial x} & =\frac{2\pi a_{f}\omega_{f}\Delta \tilde{\chi }\left(\omega_{0}\right)}{c}\omega \left\{\frac{\partial }{\partial \omega }+i\frac{2n^{\prime }x}{c}\left[\omega -\omega_{\text{pump}}\right]\right\}\\ & \;\times \tilde{A}\left(x,\omega \right). \end{array}$$

Equation (17) can be solved analytically via the method of characteristics for any input field $\tilde{A}\left(x=0,\omega \right)={\tilde{A}}_{0}\left(\omega \right)$ as

(18) $$\begin{array}{ll} \tilde{A}\left(x,\omega \right) & ={\tilde{A}}_{0}\left(\omega s\left(x\right)\right)\\ & \;\times {\text{e}}^{\text{i}\frac{2n^{\prime }\left(\omega_{0}\right)}{c}\omega \left\{\frac{\omega }{4\gamma }\left[s{\left(x\right)}^{2}-1-2\gamma x\right]-\frac{\omega_{p}}{\gamma }\left[s\left(x\right)-1-\gamma x\right]\right\}}, \end{array}$$

where the scaling parameter *s*(*x*) is defined as

(19) $$s\left(x\right)={\text{e}}^{\gamma x}\;$$

with

(20) $$\gamma =2\pi \Delta \tilde{\chi }\left(\omega_{0}\right)a_{f}\omega_{f}/c,$$

which is an inverse characteristic length defined by the gradient of the refractive index modulation. Reinserting our analytical solution (18) into Eq. (10) and still treating dispersion to the first order, we obtain the electric field *E*(*x*, *ω*) of the probe pulse, which in the co-moving frame ${\tilde{E}}_{\text{co}}\left(x,\omega \right)=\tilde{E}\left(x,\omega \right){\text{e}}^{-\text{i}\frac{\omega x}{v_{\text{pump}}}}$ is given by

(21) $${\tilde{E}}_{\text{co}}\left(x,\omega \right)={\tilde{E}}_{0}\left(\omega s\left(x\right)\right){\text{e}}^{\text{i}\frac{2n^{\prime }\left(\omega_{0}\right)}{c}\omega \left\{\frac{\omega }{4\gamma }\left[s{\left(x\right)}^{2}-1\right]-\frac{\omega_{p}}{\gamma }\left[s\left(x\right)-1\right]\right\}},$$

where ${\tilde{E}}_{0}={\tilde{A}}_{0}$ is the electric field at the input.

Equation (21) shows that the modification of the probe pulse depends directly on the scaling parameter *s*(*x*), Eq. (19), which, in turn, depends on the temporal slope of the refractive index. Therefore, for positive or negative sign of the slope, the scaling parameter either falls or grows (respectively) in an exponential manner as the pulse propagates.

## 4 Solution for a Gaussian pulse

To illustrate these effects, we consider as an input at *x* = 0 an initially Gaussian probe pulse of width *σ*_0_, centered at time *t*_0_, with a carrier frequency *ω*_0_. In the co-moving frame, the propagating pulse at the input can be written as

(22) $${\tilde{E}}_{0}\left(\omega \right)=e^{-\frac{{\left(\omega -\omega_{0}\right)}^{2}}{2\sigma_{0}^{2}}}{\text{e}}^{-\text{i}t_{0}\omega }.$$

For this initial pulse, Eq. (21) predicts the pulse to change during its propagation as

(23) $$\begin{array}{ll} {\tilde{E}}_{\text{co}}\left(x,\omega \right) & =e^{-\frac{{\left(\omega -\omega_{0}/s\left(x\right)\right)}^{2}}{2{\left(\sigma_{0}/s\left(x\right)\right)}^{2}}}e^{-it_{0}s\left(x\right)\omega }\\ & \;\times {\text{e}}^{\text{i}\frac{2n^{\prime }\left(\omega_{0}\right)}{c}\omega \left\{\frac{\omega }{4\gamma }\left[s^{2}\left(x\right)-1\right]-\frac{\omega_{\text{pump}}}{\gamma }\left[s\left(x\right)-1\right]\right\}}. \end{array}$$

Equation (23) describes the spectral shape of a probe pulse *E*(*x*, *t*) co-propagating in the linear regime of a temporal modulation in 1D and predicts several effects controlled by the exponential scaling parameter *s*(*x*), which describes the space-time warp experienced by the probe [24].

In the case of a negative temporal modulation slope, as depicted in Figure 2, it predicts the probe to be spectrally broadened, blue-shifted and amplified. If the probe is placed on the oppositely-signed slope the effects are reversed, leading to a spectrally narrowed, red-shifted and weakened pulse. All of the aforementioned effects are encoded in the first exponent in Eq. (23).

The central frequency of the probe pulse changes with propagation length as *ω*_0_/*s*(*x*). When the probe is placed on the negative slope of the refractive index modulation, *s*(*x*) reduces with *x*, leading to the blue shift of the central pulse frequency, as expected from the simple phase modulation. The nonlinear *x*-dependence of *s*(*x*) describes deviations from this simple behavior as the space-time warp develops exponentially.

The spectral broadening and the pulse amplification are encoded in the change of the Gaussian width *σ*_0_/*s*(*x*), which scales the same way as the induced frequency shift. The complex phase factors in the second and third exponents represent the two sources of frequency chirp.

The second exponent in Eq. (23) shows that the initial delay *t*_0_ between the probe and the revival center scales with *s*(*x*), independently of dispersion. The change in delay means that the probe moves towards or away from the center of the revival, depending on the sign of the spatio-temporal slope, defining the character of the space-time warp [24].

The phase factor in the third exponent in Eq. (23) primarily describes the dispersion-induced spatio-temporal drift of the probe relative to the revival, as well as the accumulated frequency chirp. Analysis of Eq. (23) shows that for sufficiently short distances, where *s*(*x*)-dependent terms can be approximated as linear functions of *x*, the dispersion-induced drift can be related to difference of group velocities of the pump and the probe pulses.

The overall behavior of the modified pulse in the time domain predicted by the frequency-domain expression Eq. (23) has physically transparent and intuitive origin. We consider the pump and probe to share the same initial wavelength, and therefore velocity, and let us place the probe at the center of the negative revival slope, setting *t*_0_ = 0 in Figure 2. This corresponds to a pump-probe delay of 4.425 ps in the laboratory frame.

In this case, as the probe “rides” on the time-dependent refractive index, the front and the back of the pulse experience different refractive indices: the front sees a higher refractive index and the back sees a lower one. The front propagates slower and the back propagates faster, resulting in a uniformly compressed and blue-shifted pulse.

Dispersion comes into play as a secondary effect limiting the process as it slows the probe down due to the increasing blue shift and gradually moves the probe out of the temporal window with dominating linear modulation of the refractive index.

The blue shift of the probe leads to light amplification proportional to the increasing photon energy. The microscopic origin of the blue shift is the stimulated anti-Stokes Raman scattering [25], which increases the newly-generated frequency by combining the energy of the incident photon with the rotational (or vibrational) molecular energy, in a process repeated in a cascade fashion [26–28]. Clearly, the number of photons in the pulse is conserved throughout the process, see e.g. recent work [7], as has been also understood in the context of electro-optical modulation [1]. Hence, the increase in the total energy of the pulse is directly proportional to the accrued frequency shift.

Last but not least, Eq. (23) shows that the peak field strength of the pulse in the time domain also increases for decreasing *s*(*x*), roughly as 1/*s*(*x*), with the peak intensity growing as 1/*s* ^2^(*x*).

## 5 Results

To validate the intuition gained from our theoretical analysis, we compare the analytical solution (23) with a numerical simulation of Eq. (14). For the medium we choose nitrogen gas at atmospheric conditions. The pump pulse has peak intensity of 3.3 × 10^13^ W/cm^2^ and a full width of 100 fs. The dispersion relation *n*(*λ*) is approximated by the Sellmeier equation [29]:

(24) $$n\left(\lambda \right)=1+\frac{1.9662731}{22086.66-\lambda^{2}}+\frac{2.7450825\times 10^{-2}}{133.85688-\lambda^{2}},$$

where the wavelength *λ* is measured in angstroms.

**Figure 3:**
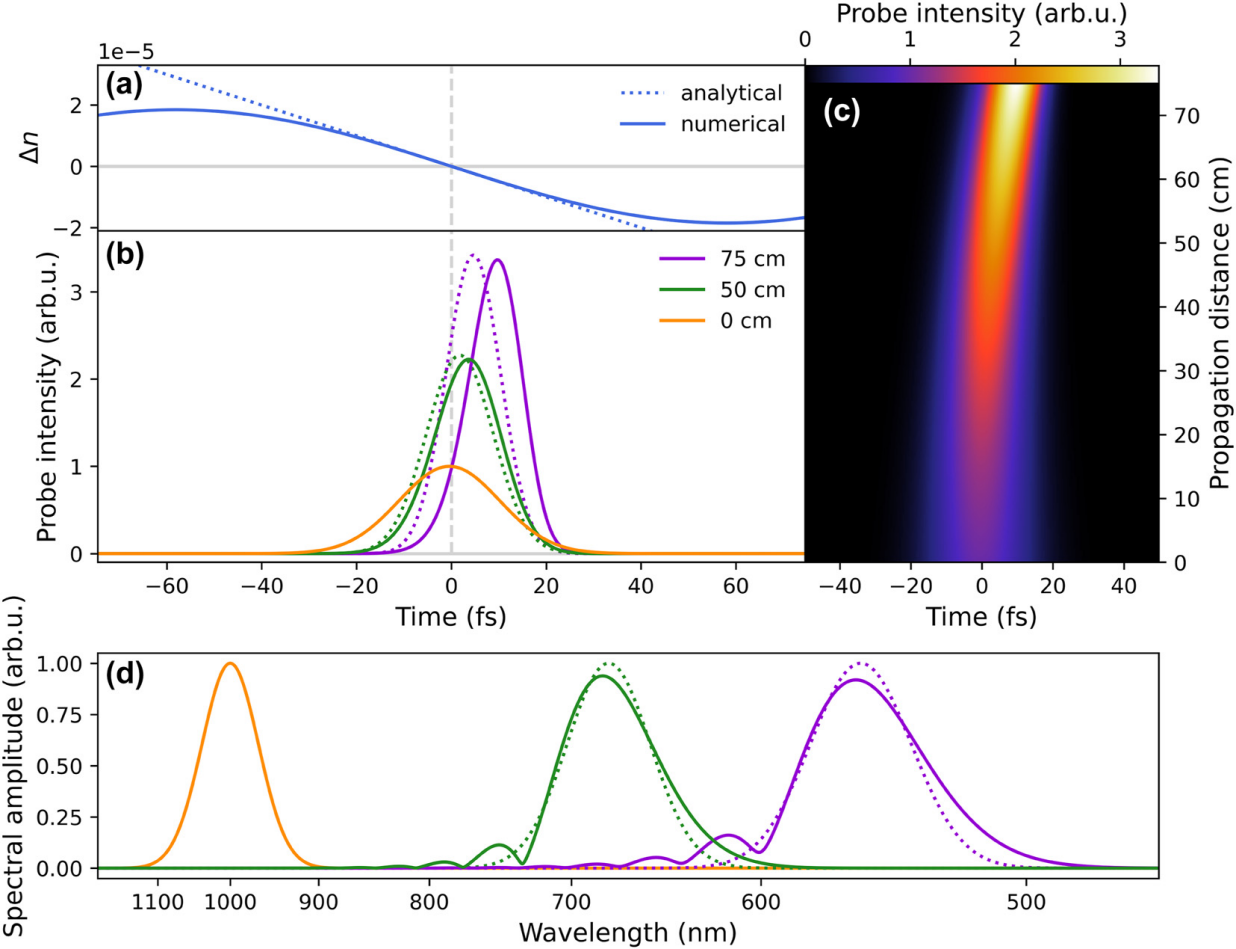
Propagation of the probe pulse initially positioned in the center of (a) a co-moving odd rotational revival. (b, c) Intensity of the probe pulse during propagation. (d) Spectral amplitude of the initial probe field (orange) and after 50 cm (green) and 75 cm (purple) propagation distance. Analytical result corresponds to Eq. (23) (dotted line) and computational (solid line) result is obtained through numerical calculation of Eq. (14).

Results of the simulations are presented in Figure 3. Panel (a) shows the relevant part of the refractive index induced by the pump. Panel (c) shows the modification of the probe pulse as a function of propagation distance. Panel (b) presents the envelope of the probe pulse in the beginning of the propagation as well as after 50 cm and 75 cm of propagation, where the analytical solution (23) is represented by dashed lines while the numerical solution obtained by solving Eq. (14) through an iterative finite-difference method is represented by the solid line. We can see that the probe pulse is compressed in time and amplified as it propagates, while gradually slowing down relative to the modulation function and steepening towards the back. Meanwhile the spectrum of the probe gets heavily blue-shifted and broadened, see panel (d). After 75 cm of propagation the central wavelength has been shifted from the near IR deep into the visible while the peak field strength is increased by a factor of 1.8, corresponding to over a factor 3 increase in the intensity.

Our analytical solution (23) shown with dotted lines in Figure 3(b, d) exhibits only minor deviations from the computational result (14). The larger deviation between analytical and numerical results appears in the temporal domain affecting the delay of the pulse while its shape changes slightly. These differences are mostly attributed to the neglect of quadratic and higher order terms in the expansion of the frequency dispersion. In the spectral behavior, the results of the two approaches agree well for the central frequency of the propagating pulse, and diverge for its bandwidth (also the analytical approach does not describe fringes). This difference is also associated with the neglect of quadratic and higher terms, but in this case mostly due to the approximation of the temporal modulation function.

## 6 Conclusions

Our results outline a novel mechanism of pulse compression, amplification and frequency conversion, which has strong parallels with the mechanism recently described in [8], but deals with completely different media and takes realistic account of medium dispersion in a consistent manner. In the process, we have addressed the conceptual problem of combining time and frequency domains, which is necessary when dealing with dispersive media that experience time-dependent modulations of their properties.

While pulse compression by molecular modulators has been known for about two decades [20, 21, 26, 27] in the ultrafast optics community, to the best of our knowledge, the pulse compression mechanism described here has been overlooked by this community. The physics underlying this compression mechanism is very simple: when placed on a linear slope of the refractive index, the front and the rear of the probe pulse move with different speeds, leading to pulse compression with blue shift or pulse stretching with red shift, depending on the sign of the slope. Simultaneously, the pulse is either amplified or weakened. While the number of photons in the pulse is conserved, the energy grows or decreases proportionally to the shift of the carrier frequency. The peak field strength also grows (decreases) proportionally to the pulse compression (stretching).

Crucially, our analytical solution demonstrates that all three effects, pulse compression, pulse amplification and frequency conversion, are exponential. The exponential growth of these effects together is only limited by dispersion. Our numerical simulations show that substantial amplification, compression and frequency conversion can be observed under experimental conditions that we, as theorists, dare classify as routine.

## Supplementary Material

Supplementary Material Details
