# Supplementary material for: Controlled compression, amplification and frequency up-conversion of optical pulses by media with time-dependent refractive index
Source: Nanophotonics. 2023 Mar 9;12(14):2921–8. doi: 10.1515/nanoph-2022-0818 (PMC11502027; doi:10.1515/nanoph-2022-0818)
Supplement: Supplementary file 1 — Supplementary Material Details [file j_nanoph-2022-0818_suppl_001.pdf]

Research Article

Alexander G. Löhr*, Misha Y. Ivanov, and Margarita A. Khokhlova

Controlled compression, amplification and frequency up-conversion of optical pulses by media with time-dependent refractive index. Supplementary material

https://doi.org/10.1515/sample-YYYY-XXXX
Received Month DD, YYYY; revised Month DD, YYYY; accepted Month DD, YYYY

In order to check the validity of our analytical method, we apply our model to the experimental conditions used in F. Noak and co-authors [1].

The experiment in question uses a strong fundamental of 50 fs duration at 800 nm to induce molecular rotations in a 25 cm hollow-core waveguide filled with nitrogen gas at 0.5 bar and ambient conditions. Then a delayed third harmonic of the fundamental at 268 nm propagates through the rotationally-excited medium and the change in its spectral composition at the end of the waveguide is measured for different initial pump-probe delays. The energy of the pump at the exit of the 128 μm diameter waveguide is measured at 100 μJ, while the energy of the probe remains below 1 μJ. This clearly fulfills the criteria of our model to have a significantly stronger pump than probe.

To model the shape of the pump-induced rotational revivals, we use the simplified alignment model derived in [2], which has proven to approximate the rotational revivals predicted by the more sophisticated analytical alignment model introduced by Leibscher et al. [3] very well.

Using the theory introduced in [2], we can describe the first major alignment revival (roughly 4 ps delayed from the pump pulse) induced by the pump as:

$$\langle \cos^2(\theta) \rangle (t) = \frac{1}{3} - A \sin\left(B \frac{\hbar}{I} t \right) e^{-\frac{3}{2}\left(\frac{\hbar}{I}\right)^2 \sigma^2 t^2} , \qquad (1)$$

where the coefficients A, B are given by

$$A = \frac{log_2(3) P}{B} ,$$
$$B = \sqrt{3P^2 + 9\sigma^2 + 4} \qquad (2)$$

with the moment of inertia $I \approx 1.4 \times 10^{-39}\mathrm{gcm}^2$, taken from [4], of the nitrogen molecule, in the ground state. Further the system parameters σ and P are defined analogous to [3] and depend on the temperature T and the pump intensity I_P through

$$\sigma^2 = \frac{k_B T I}{\hbar^2} ,$$
$$P = \frac{\Delta\alpha}{2\hbar c \epsilon_0} \int_{\infty}^{\infty} I_P(t) dt . \qquad (3)$$

Here k_B is the Boltzmann constant, $\Delta\alpha = \alpha_{\parallel} - \alpha_{\perp} \approx 8 \times 10^{-41}\,\mathrm{C} \cdot \mathrm{m}^2/\mathrm{V}$, taken from [5], the difference in parallel and perpendicular polarizabillity, c the speed of light in a vacuum and ϵ_0 the vacuum permittivity.

Assuming the gas temperature to be 300 K, the pump energy to be spread evenly over the fiber cross-section, and using all abovementioned experimental parameters, we obtain from (3) $\sigma \approx 10$ and $P \approx 1.1$ (note that it is not 2 as stated in [1]).

Since the probe in this experiment is not placed in the center of the revival, we cannot use the sinusoidal approximation derived in the main text, instead we adapt our numerical routine to account for the entire convolution integral in the propagation equation (12).

The exact picosecond delay between the pump and probe pulses could not be measured in the experiment, however the delay of the probe could be controlled with femtosecond resolution. Therefore all the delays were measured relative to the delay t_0, which corresponds to the delay at which a spectral shift was first observed.

***Corresponding author: Alexander G. Löhr, Misha Y. Ivanov, Margarita A. Khokhlova,** Max Born Institute for Nonlinear Optics and Short Pulse Spectroscopy, Berlin, Germany, e-mail: alexander.loehr@physik.hu-berlin.de
***Corresponding author: Alexander G. Löhr, Misha Y. Ivanov,** Department of Physics, Humboldt University, Berlin, Germany
Misha Y. Ivanov, Blackett Laboratory, Imperial College London, London, UK
Margarita A. Khokhlova, Department of Physics, King's College London, London, UK

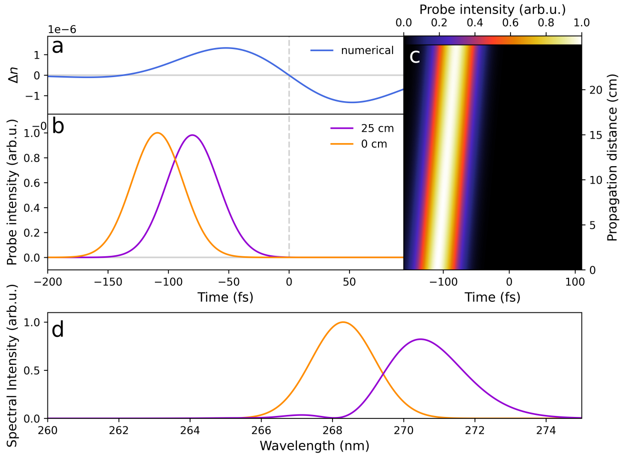

Fig. 1: Propagation of the 268 nm probe pulse initially positioned at109 fs (delay t_0 + 180 fs in [1]) before the center of (a) an rotational revival co-moving with the group velocity of the 800 nm pump. (b,c) Intensity of the probe pulse during propagation.
(d) Spectral intensity of the initial probe field (orange) and after and 25 cm (purple) propagation distance. This result is obtained through numerical solution of Eq. (12).

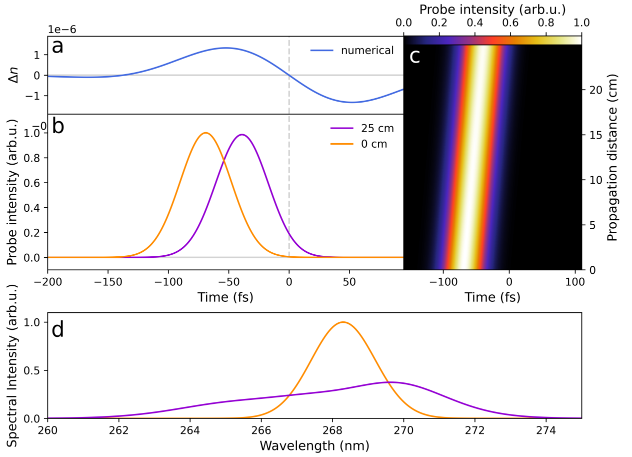

Fig. 2: Same as Fig. 1 for the probe at 69 fs (delay t_0 + 180 fs).

Setting this non-measured initial delay t_0 to 249 fs, we are able to replicate the measured spectral changes very reasonably for the delays measured in the experiment, see Fig. 1 - 4.

The successful application of our analytical method makes us confident in the accuracy of the calculations performed in the main body of this work.

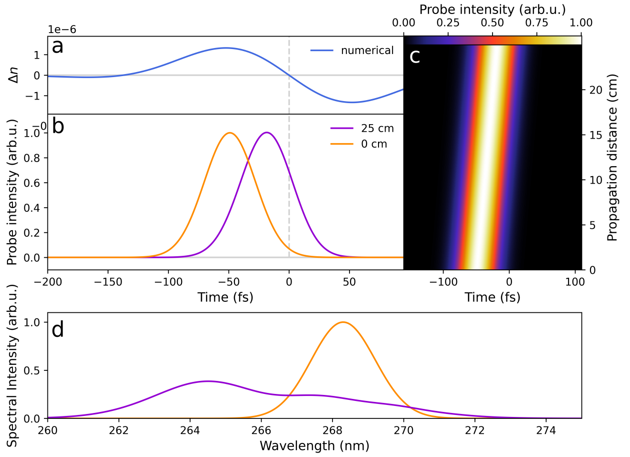

Fig. 3: Same as Fig. 1 for the probe at 49 fs (delay t_0 + 200 fs).

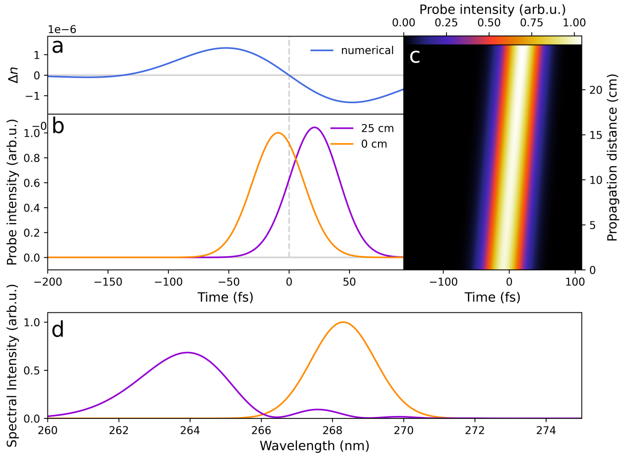

Fig. 4: Same as Fig. 1 for the probe at 9 fs (delay t_0 + 240 fs).

References

[1] F Noack, O Steinkellner, P Tzankov, H-H Ritze, J Herrmann, and Y Kida. Generation of sub-30 fs ultraviolet pulses by raman induced phase modulation in nitrogen. *Optics Express*, 13(7):2467–2474, 2005.

[2] A Loehr, M Khokhlova, and M Ivanov. A simple analytical alignment model for laser-kicked molecular rotors. *in preparation.*

[3] M Leibscher, I Sh Averbukh, and H Rabitz. Enhanced molecular alignment by short laser pulses. *Physical Review A*, 69(1):013402, 2004.

[4] Klaus-Peter Huber. *Molecular spectra and molecular structure: IV. Constants of diatomic molecules*. Springer Science & Business Media, 2013.

[5] K M Gough, M M Yacowar, R H Cleve, and J R Dwyer. Analysis of molecular polarizabilities and polarizability derivatives in H2, N2, F2, CO, and HF, with the theory of atoms in molecules. *Canadian journal of chemistry*, 74(6):1139–1144, 1996.
